# Low Incubation Temperature During Late Incubation and Early Feeding Affect Broiler Resilience to Necrotic Enteritis in Later Life

**DOI:** 10.3389/fvets.2021.784869

**Published:** 2021-12-14

**Authors:** Hendrikus J. Wijnen, Carla W. van der Pol, Inge A. M. van Roovert-Reijrink, Joren De Smet, Aart Lammers, Bas Kemp, Henry van den Brand, Roos Molenaar

**Affiliations:** ^1^Adaptation Physiology Group, Department of Animal Sciences, Wageningen University & Research, Wageningen, Netherlands; ^2^Research Department, HatchTech B.V., Veenendaal, Netherlands; ^3^Clinical Research Organization, Poulpharm BVBA, Izegem, Belgium

**Keywords:** broiler, incubation, eggshell temperature, early feed, necrotic enteritis

## Abstract

Resilient animals can cope with environmental disturbances in life with minimal loss of function. Resilience can be enhanced by optimizing early-life conditions. In poultry, eggshell temperature (**EST**) during incubation and early feeding are two early-life conditions that are found to alter neonatal chick quality as well as immune response in later life. However, whether these early-life conditions affect disease resilience of chickens at later ages has never been studied yet. Hence, we studied the effects of EST [(37.8°C (**control**) or 36.7°C (**lower**)] during late incubation (≥embryonic days 17–19.5) and feeding strategy after hatch [immediately (**early feeding**) or 51–54 h delayed (**delayed feeding**)] on later-life broiler resilience in a 2 × 2 factorial arrangement. At hatch, 960 broilers of both sexes from a 54-week-old Ross breeder flock were equally divided over 32 pens (eight replicate pens per treatment combination) and grown for 6 weeks. Necrotic enteritis was induced by a single inoculation of *Eimeria* spp. at d 21 and repeated *Clostridium perfringens* inoculation (3×/d) during d 21–25. Mortality and body weight (BW) gain were measured daily during d 21–35 as indicators of resilience. Additionally, disease morbidity was assessed (gut lesions, dysbacteriosis, shedding of oocysts, footpad dermatitis, and natural antibody levels in blood). Results showed a lack of interaction between EST and feeding strategy for the vast majority of the variables. A lower EST resulted in lower BW gain at d 5 and 8 post *Eimeria* inoculation (*P* = 0.02) and more *Eimeria maxima* oocysts in feces at d 8 post *Eimeria* inoculation compared to control EST (*P* < 0.01). Early feeding tended to lower mortality compared to delayed feeding (*P* = 0.06), but BW gain was not affected by feeding strategy. Morbidity characteristics were hardly affected by EST or feeding strategy. In conclusion, a few indications were found that a lower EST during late incubation as well as delayed feeding after hatch may each impair later-life resilience to necrotic enteritis. However, these findings were not manifested consistently in all parameters that were measured, and conclusions are drawn with some restraint.

## Introduction

There is an increased global concern about the high use of antimicrobials and potentially related resistance threats (1). Efforts are made in various areas to reduce the usage of antibiotics. In animal husbandry, this reduction in antibiotics usage led to a rising interest in alternative approaches to enhance animal health, e.g., by enhancing animal resilience. Resilience can be defined as the capacity of an animal to absorb environmental disturbances and reorganize with minimal loss of function (2). In animal husbandry, environmental disturbances can be for instance changes in social structures, thermal conditions, or disease outbreaks. When focusing on the latter one, a resilient animal has a lower chance to become ill, and once it does become ill, it will show rapid recovery. Consequently, increased animal resilience may lead to a lower need of antibiotics, improved animal welfare, and beneficial revenues and sustainability. Resilience of animals is likely affected by early-life conditions, because critical windows for immune organ development exists during early life (3) and environmental conditions in this period can affect the animal's immune system in later life (4).

In poultry, one environmental condition during early life that may affect chicken resilience is the embryo temperature during incubation. Embryos act poikilotherm, and therefore, their body temperature and, as a result, their metabolic rate and development are affected by their temperature. Embryo temperature is accurately reflected by eggshell temperature (**EST**) (5). Changes in EST patterns during incubation can affect chick quality at hatch and can even affect growth performance throughout life (6–10). Moreover, EST can affect the immunocompetence of broilers in later life by alterations in peripheral lymphocyte numbers (11), mucin expression and salmonella enteritidis colonization in the gut (12), and Newcastle disease vaccination response (13). So far, a constant EST of 37.8°C throughout incubation is regarded optimal in terms of chick quality at hatch (14). However, there are some indications that lowering EST to 36.7°C during late incubation may benefit embryo development in terms of yolk-free body mass (15) and heart weight (15, 16). However, other aspects of embryo development, such as chick length or bursa and intestine development, may be impaired by a lower EST during late incubation (13, 16). Unfortunately, none of these studies investigated post-hatch broiler disease resistance, and therefore, possible effects of a lower EST during late incubation on broiler resilience against diseases in later life are unknown.

Another early-life condition that may affect broiler resilience in later life is the provision of feed and water immediately after hatch moment (referred to as “early feeding”). Currently, chicks are often withheld from feed and water during the period from hatch moment at the hatchery until placement at a farm. The duration of this period varies between batches of chicks, but likely ~48 h is common whereas it was described to last up to 72 h in case of long transport duration (17, 18). Despite the availability of abdominal residual yolk during this period, withholding neonatal chicks from feed and water has been shown to result in body weight (BW) loss and an impaired or delayed onset of gastrointestinal development (19–22), thermoregulation (23), and immunocompetence (24–26). Feeding chicks in this period will thus affect neonatal chick development but is also known to have lasting effects on performance, mortality, and immune response after the period of feed deprivation (24, 27–29). Most studies related to early feeding provided vaccines or model antigens to induce a disease response rather than inducing a disease. Therefore, based on these studies, no definite conclusion can be drawn on whether possible alterations in immune response found by model antigens result in altered broiler resilience. The use of disease models in studies could elucidate this, especially if kinetics in functional losses or pathogenesis are determined. Until now, such studies are very limited (30, 31).

This study aimed to investigate the effects of EST during late incubation and feeding strategy immediately post hatch on later-life resilience. Both factors may interact with each other as EST may for instance affect intestinal morphology and digestive enzyme activity in such a way that a chick is better prepared for exogenous feed intake at the hatch moment and will have a higher benefit from early feeding.

## Materials and Methods

This study was conducted during April to May 2019 at Wageningen University & Research, The Netherlands (incubation and hatch period), and Poulpharm, Zwevegem, Belgium (grow-out period). The experimental protocol from hatch until transport of day-old chicks was approved by the Governmental Commission on Animal Experiment, The Hague, The Netherlands, approval number: 2018.W-0020.001. The experimental protocol from transport until the end of the experiment was carried out according to the recommendations and following approval of the Ethical Committee of Poulpharm, Belgium, approval number P19034-FP.

### Experimental Design

The experiment was set up as a 2 × 2 factorial arrangement with EST during late incubation and feeding strategy after hatch as treatments. EST from embryonic day (**E**) 17 until E19.5 was set at 37.8°C (**control**) or at 36.7°C (**lower**), whereas feeding strategy included access to feed and water within 3–6 h after hatch (**early feeding**) or within 51–54 h after hatch (**delayed feeding**).

### Egg Selection

Eggs from a 54-week-old Ross 308 broiler breeder flock were stored at a commercial hatchery (Lagerwey BV, Lunteren, The Netherlands) for 4 days at 20°C. All eggs were laid on the same date in two broiler houses at one farm. Both houses were held under similar environmental and management conditions, and eggs coming from both houses were randomly mixed between treatments. To exclude potential effects of initial egg weight (32), 10 egg trays with 150 eggs each were bulk-weighed to determine the average egg weight (70.1 g). Three equal weight classes within 1.5 g of the average egg weight were determined (69.35–69.85, 69.86–70.35, and 70.36–70.85). Thereafter, eggs were weighed individually until 446 first-grade eggs (clean and without hairline cracks or malformations) per weight class were selected (total 1,338 eggs). Eggs were transported for ~30 min to the animal research facility of Wageningen University & Research (Wageningen, The Netherlands), where eggs of each weight class were equally divided over 16 setter trays (type 88 Setter Tray, HatchTech, Veenendaal, The Netherlands).

### Incubation

All 16 trays were set in one incubator [type climate respiration chamber, details provided by (33)]. Four EST sensors (NTC Thermistors: type DC 95; Thermometrics, Somerset, UK) were attached to the equator of the eggshell of four individual eggs, using a silicone heat sink compound (Type 340; Dow Corning, Midland, MI) and a small piece (~1.5 × 1.5 cm) of elastic performance tape (Leukotape K, Essity, Hamburg, Germany). A 22-h preincubation warming profile was applied before the onset of incubation, adapted from (34) such that eggs were linearly warmed from storage temperature to 30.6°C EST in 5 h and from 30.6 to 37.8°C EST in 17 h. The moment eggs reached an EST of 37.8°C was considered to be the start of incubation (E0). Until E17, the incubator temperature was continuously adjusted, based on the median temperature of the four EST sensors to aim at an EST of 37.8°C. Eggs were turned every hour by an angle of 45° from horizontal. Relative humidity (RH) was maintained between 50 and 55%, and CO_2_ level was maintained below 3,500 ppm.

At E17, all eggs were candled, and eggs containing a viable embryo (*N* = 1,072; 86.7% of fertile eggs at set) were transferred to hatching baskets. Eggs were transferred to one hatcher basket per setter tray. These hatcher baskets were divided over four incubators (four baskets per incubator). Two incubators were aimed at an EST of 37.8°C (control EST), whereas the other two incubators aimed at an EST of 36.7°C (lower EST). EST control in all four incubators was performed as described above. RH was maintained between 45 and 75%, and CO_2_ levels were maintained below 3,500 ppm. After 468 h of incubation (E19 12 h), the incubator temperatures were fixed at their actual setting, and EST was allowed to change as chicks started to emerge from the eggshell. Details about actual EST are provided in (35).

### Hatch and Early Feeding

From 468 h of incubation onward, every 3 h, the incubators were opened to check whether chicks had hatched. A chick that was fully emerged from the shell was marked with a colored dot on the head using a permanent marker. After marking, chicks were placed back in their original hatcher basket to dry. Three hours after a chick was marked, it was pulled from the incubator and classified either as a second-grade chick if any abnormality was observed (e.g., crossed beak, blindness, exposed brains, four legs, and exposed yolk) or as a first-grade chick (all remaining chicks). All second-grade chicks were excluded from the experiment. The first-grade chicks (*N* = 1,028) were feather sexed, marked with a plasticized paper neck tag (size 5 × 2 cm), and transferred to HatchCare hatcher baskets (HatchTech, Veenendaal, The Netherlands). In half of these baskets (early feeding treatment), *ad libitum* starter pellet feed (details given in the “Grow-out” section below) and fresh water were provided. The other half of these baskets had empty gutters and troughs (delayed feeding treatment). Chicks were stored in these hatcher baskets in a chick storage room at 36.0°C and 55% RH without forced airflow until E21 12 h. At E21 12 h, all water gutters were emptied and baskets (including residual feed in baskets from early feeding treatment) were transferred to a climate-controlled van designed for chicken transportation at 29.4°C and 36% RH with forced airflow (Chickliner, Renswoude, The Netherlands). Chicks were transported during ~3 h to a grow-out facility from Poulpharm, Zwevegem, Belgium.

### Grow-Out

#### Layout

On arrival at the grow-out facility (regarded as day 0), 960 broiler chicks were selected randomly and divided over 32 floor pens in one broiler house (eight replicate pens per treatment). Pens were divided over eight equal blocks (four pens per block), and all four treatments were allocated to each block. At placement, each pen contained 15 male and 15 female broilers. Pen size was 260 × 105 cm and fenced with a 60-cm-high solid mesh. The concrete pen floor was covered with a 1-cm-thick layer of wood shavings. Each pen contained four drinking nipples and one metal feed silo (35-cm diameter).

#### Delayed Feeding

At placement, pens from the delayed feeding treatment were temporarily divided with a fence in the middle, which divided the pen into an unfed side and a fed side. The unfed-side floor was covered with cardboard to prevent litter consumption. At placement, all broilers within these pens were positioned in the unfed side of the pen. Thereafter, each broiler was relocated individually to the fed side of the pen 48 h after it had received its neck tag. As a result, delayed-fed broilers had access to feed and water within 51–54 h post hatch. Fences and cardboard were removed after all broilers were relocated to the fed side of the pen. Which treatment was contained in a pen was blinded from that moment onward.

#### Feed and Vaccinations

A starter pelleted diet (ME broiler = 2,838 kcal/kg, CP = 221.8 g/kg, digestible lysine = 12.9 g/kg) with a diameter of 2.6 mm was provided from d 0 until d 13, a grower pelleted diet (ME broiler = 2,999 kcal/kg, CP = 269.4 g/kg, digestible lysine = 15.17 g/kg) with a diameter of 3.2 mm was provided from d 13 until d 28, and a finisher pelleted diet (ME broiler = 3,001 kcal/kg, CP = 188.6 g/kg, digestible lysine = 10.85 g/kg) with a diameter of 3.2 mm was provided from d 28 onward. Diets did not contain coccidiostats and were produced by Research Diet Services (RDS, Wijk bij Duurstede, The Netherlands) according to the guidelines of the Federation Dutch Animal Feed chain (36). Feed and water were provided *ad libitum*. At d 0, broilers were spray vaccinated against Newcastle disease (Avishield ND) and infectious bronchitis (Poulvac IB primer). At d 12, Newcastle disease vaccination (Avishield ND) and Gumboro disease vaccination (Nobilis Gumboro D78) were provided *via* drinking water.

#### Climate

Ambient temperature set point was 35°C at placement and was linearly decreased to 20°C at d 24, and this set point was maintained until the end of the study. As a difference in ambient temperature preference was expected between early- and delayed-fed broilers, a heat lamp was provided in the middle of each pen from placement until d 12, meaning that each broiler could choose its own preferred ambient temperature. RH was on average 34.5% and varied between 20 and 50%. At placement, 24 h of light was provided, and from d 2 onward, 1 h of darkness every 24 h was added each day until 6 h of darkness every 24 h was provided by d 7, and this lighting schedule was maintained until the end of the study.

### Necrotic Enteritis Model

A subclinical necrotic enteritis (**NE**) was induced by adapting a protocol from (37). Fish meal (10%) and rye (5%) were included into the grower diet (d 13–28) as predisposing factors for NE induction. At d 21, all broilers were orally inoculated with 1-ml inoculum containing field isolates of *Eimeria acervulina* (80,000 oocysts), *Eimeria maxima* (40,000 oocysts), and *Eimeria mitis* (5,800 oocysts). These field isolates were purified and differentiated out of samples from Germany, Australia, and Germany, respectively. At the day of *Eimeria* inoculation and 4 days thereafter (d 21–25), all broilers were also orally inoculated 3×/day (~8:00 am, 11:00 am, and 02:00 pm) with ~1 × 10^9^ colony-forming units of a *netB*-positive *Clostridium perfringens* strain 56 (38, 39). The *C. perfringens* strain was streaked onto a Columbia sheep-blood agar (37°C, anaerobic incubation for ~18 h). Next, different isolated colonies were selected for further constitution of the bacterial inoculum. Colonies were aseptically transferred into a brain heart infusion (BHI) medium and incubated overnight (37°C, anaerobic incubation for ~15 h). The number of CFU/ml in the final inoculum composition was calculated based on viable cell counts, after streak plating a 10-fold dilution series of the inoculum onto Columbia sheep-blood agar. All plates were incubated anaerobically at 37°C for ~18–24 h.

### Data Collection

The response of broilers to the NE induction was monitored from the day of *Eimeria* inoculation (d 21) until the end of the experiment (d 35) by determining mortality and BW gain as indicators of resilience. Disease morbidity was assessed by determining NE lesions, coccidiosis, dysbacteriosis, oocyst shedding, natural antibody levels in blood, and footpad dermatitis.

Mortality was determined by daily monitoring of dead broilers each morning from the day of *Eimeria* inoculation until the end of the experiment. Broilers were observed daily to check their health and well-being and were culled by caretakers if a humane endpoint was reached, as defined by (40).

Changes in BW were measured to determine functional loss. From d 21 up to and including d 31 and once finally at d 35, all broilers were weighed individually during the morning. Average daily gain (**ADG**) was calculated for each broiler that survived during the experiment.

Necrotic lesions, coccidiosis, and dysbacteriosis were scored by dissecting broilers ~3 h before *Eimeria* inoculation and at d 6, 7, and 14 post *Eimeria* inoculation (**PEI**; respectively d 21, 27, 28, and 35 post hatch). All these were scored blind by trained veterinarians. At ~3 h before *Eimeria* inoculation, a varying number of broilers was dissected per pen such that the remaining number of broilers in each pen was 20 of equal sex ratio. Thereby, possible effects of varying animal densities between pens, caused by mortality between hatch and onset of NE, on outcome of NE response was prevented. This procedure resulted in dissection of a total of 100 broilers at ~3 h before *Eimeria* inoculation. To ensure that the remaining living broilers (20 per pen) were closest to treatment average, broilers to be used for dissection (*n* = 100) were selected as follows. The average BW at d 14 post hatch was calculated per sex per treatment group. Subsequently, within each pen and per sex, the first broiler that was selected for dissection had the most deviating higher weight at d 14 than treatment group average. The second broiler that was selected had the most deviating lower weight than treatment group average and so on until 20 broilers per pen remained. At d 6 and 7 PEI, 10 broilers per pen (equal sex ratio) were dissected (five broilers per pen per day). This time, broilers within each pen were selected for dissection if their BW at d 25 deviated the most from the average BW of similar sex and treatment groups. At d 14 PEI, five broilers per pen were dissected (three females and two males). Broilers were selected based on their BW at d 31 as described for d 6 and 7 PEI.

Necrotic lesions were scored according to protocol (41). At d 6 and 7 PEI, all dissected broilers were scored on macroscopic NE lesions in the small intestine (duodenum to ileum) on a severity scale from 0 (no gross lesions) to 6 (diffuse necrosis). Broilers with NE lesion scores of 2 or more were considered as NE positive, and incidence of NE was based on this classification.

Coccidiosis was determined in all dissected broilers during all dissection moments according to protocol (42). *E. acervulina, E. maxima*, and *Eimeria tenella* macroscopic lesions were each scored on a severity scale 0 (no gross lesions) to 4 (severe lesions). The total mean lesion score (**TMLS**) was calculated by summing up the scores of each *Eimeria* type, resulting in a TMLS severity score between 0 and 12 (43). Incidence was calculated by classifying broilers with a TMLS of 1 or more as coccidiosis positive.

Dysbacteriosis was scored according to protocol (44). At the day of *Eimeria* inoculation and d 14 PEI, in all dissected broilers, the absence (score 0) or presence (score 1) of gut ballooning, undigested feed particles, redness, gut wall thickness, flaccidity, and abnormal lumen content was determined. Dysbacteriosis severity score was calculated by summing up all scores, resulting in a dysbacteriosis severity score between 0 and 10. Incidence was calculated by classifying broilers with a dysbacteriosis score of 3 or more as dysbacteriosis positive.

Oocyst shedding was assessed in all pens at 1 day before *Eimeria* inoculation and at d 4 and 7 PEI (d 20, 25, and 28 post hatch, respectively). Several fresh droppings (~200 g) were collected and mixed per pen. Oocysts per gram of feces (**OPG**) from *E. acervulina, E. maxima, E. mitis, E. tenella, Eimeria brunetti*, and *Eimeria necatrix/praecox* were counted according to the McMaster method (45). The *Eimeria* spp. differentiation was based on the morphometrical examination of each oocyst according to the protocol (46) performed by trained and validated parasitology lab technicians. *E. brunetti* and *E. necatrix/praecox* were not detected at any collection day and are therefore not discussed any further in this paper. One day before *Eimeria* inoculation, *E. acervulina, E. mitis*, and *E. tenella* oocysts were found in 75.0, 62.5, and 6.3% of the pens, respectively. Preliminary analysis showed that there was no effect of treatment on OPG prior to *Eimeria* inoculation nor at d 5 or 8 PEI for any of these three *Eimeria* species. The OPGs of these *Eimeria* species were excluded from the results as their presence 1 d before inoculation indicated that OPG numbers at d 5 and 8 PEI could not be attributed solely to the disease challenge.

At d 0, 6, and 14 PEI (d 21, 27, and 35 post hatch, respectively), blood was collected from the wing vein of two broilers per pen (one male and one female) randomly chosen from the ones that were selected for dissection. Blood was collected in natrium-heparinized tubes (Vacuette 4 ml FX, Greiner Bio-One), stored on ice, and plasma was collected after centrifugation at 2,000 × *g* for 10 min. Plasma was stored at −20°C until samples were analyzed for the level of IgY and IgM natural antibodies (**NAb**) though the amount of immunoglobulin binding to keyhole limpet hemocyanin (**KLH**) adjusted from the protocol of (47). Briefly, 96-well medium binding flat-bottomed plates (Greiner Bio-One, Alphen a/d Rijn, The Netherlands) were coated with 100 μl coating buffer (5.3 g/L Na_2_CO_3_ and 4.2 g/L NaHCO_3_; pH 9.6), containing either 2 or 4 μg/ml KLH (H8283, Sigma-Aldrich, St. Louis, MO, USA) for isotypes IgY or IgM, respectively. Plates were incubated at 4°C overnight, washed with tap water containing 0.05% Tween 20, and tapped dry. Plasma samples were 1:10 pre-diluted (both isotypes) with a dilution buffer [(phosphate-buffered saline (PBS; 10.26 g/L Na_2_HPO_4_·H_2_O, 2.36 g/L KH_2_PO_4_, and 4.50 g/L NaCl; pH 7.2), containing 1% horse serum and 0.05% Tween 20)]. Pre-dilutions were further diluted with a dilution buffer such that the tested plasma dilutions were 1:40, 1:160, 1:640, and 1:2,560 for both isotypes. Plates were incubated for 1.5 h at room temperature and then washed again with tap water, containing 0.05% Tween 20. Subsequently, plates were incubated for 1.5 h at room temperature with 1:20,000 dilutions of either goat anti-chicken IgG(Fc) (Bethyl A30-104P) or goat anti-chicken IgM (Bethyl A30-102P), each labeled with horse radish peroxidase (polyclonal antibodies from Bethyl Laboratories, Montgomery, TX, USA). Plates were washed again with tap water, containing 0.05% Tween 20, after which antibodies to KLH were visualized by adding a 100 μl substrate buffer (tetramethylbenzidine + 0.05% H_2_O_2_). After 20 min, the reaction was stopped with 50 μl of 1.25 M H_2_SO_4_, and extinctions were measured at 450 nm with a microplate spectrophotometer (Multiskan Go, Thermo Scientific, Breda, The Netherlands). Antibody titers were calculated based on log2 values of the dilutions that gave extinction closest to 50% of E_MAX_, where E_MAX_ represents the mean of the highest extinction of the standard positive plasma samples, thereby partly correcting for plate-to-plate differences (48).

Footpad dermatitis (**FPD**) for both feet was assessed in all dissected broilers at all days, using the protocol of (49). Macroscopic lesions were scored blind by trained veterinarians at 0 (no lesions, only mild hyperkeratosis, and no discoloration or scars), 1 (mild lesions, superficial lesions, erosions, papillae, and discoloration of the footpad), or 2 (severe lesions, deep lesions, ulcers, and scabs). The highest score from both feet was noted as the final score. A score of 2 was only found once (d 35). Therefore, scores of 1 and 2 were merged as a score of 1 and classified as FPD positive.

### Statistical Analyses

All data were analyzed, using the statistical software package SAS (Version 9.4, SAS Institute, 2010). The basic model used for all data was


(1) $$\begin{array}{l} {\text{Y}}_{\text{ij}}=\mu +{\text{EST}}_{\text{i}}+{\text{FEED}}_{\text{j}}+\text{EST}\times {\text{FEED}}_{\text{ij}}+{\text{e}}_{\text{ij}} \end{array}$$

where Y_ij_ = the dependent variable, μ = the overall mean, EST_i_ = eggshell temperature during late incubation (i = 36.7 or 37.8°C), FEED_j_ = feeding strategy (j = early or delayed), EST × FEED_ij_ = the interaction between EST during late incubation and feeding strategy, and e_ij_ = the error term.

Pen was considered as the experimental unit for OPG and mortality data. Preliminary analysis showed that survival lines in a Cox proportional hazard model (PhReg) crossed, and thus, assumptions of overall homogeneity of survival distributions were not met. Alternatively, the percentage of broilers that died or culling percentage was calculated per pen by summing up the number of broilers that died or were culled PEI divided by the number of broilers before *Eimeria* inoculation (20 per pen) minus the number of broilers dissected for morbidity scores at d 6 and 7 PEI (10 per pen). Total mortality was calculated as the sum of broilers that died PEI and were culled PEI.

All remaining data were collected for individual broilers, but pen was still considered as the experimental unit by extending model 1 with pen (1–32) as a random factor. Sex was added to model 1 as a fixed factor. Besides, sex-and-treatment interactions were added (sex × EST, sex × FEED, and sex × EST × FEED). Preliminary statistical analysis did not show significant effects of sex × EST or sex × EST × FEED for any of the variables, and therefore, these interactions were deleted from the model. A significant interaction between sex × FEED was found only for FPD at d 6 PEI, and sex × FEED was therefore added to model 1 for this single dependent variable only. In all other cases, interactions between sex and FEED were also excluded from the model.

The PROC MIXED procedure was used to analyze mortality data (died, culled, and total mortality), changes in BW (BW and ADG), OPG, and natural antibodies. Model assumptions were verified by inspection of residual plots, and non-normally distributed data were log-transformed. For BW during d 0 to 10 PEI, the broiler was the repeated subject, and model 1 was extended with day and the interactions between day and EST, day and FEED, and day and sex. The covariance structure was selected based on the best model fit according to the smallest Akaike's information criteria, resulting in a Toeplitz covariance structure. Data are expressed as LSmeans ± SEM. Tukey adjustments for multiple comparisons were used to compare least square means. A *P*-value ≤ 0.05 was considered as significant, and *P*-values >0.05 and ≤ 0.10 were considered as a tendency.

The PROC GLIMMIX procedure was used to analyze severity and incidence of NE lesions, coccidiosis, dysbacteriosis, and FPD. Severity scores were analyzed with a multinomial cumulative logit link function in model 1. Incidence was analyzed with a binary logit link function in model 1. For NE incidence, day (6, 7, and 14 PEI) was added as a fixed factor to model 1 and the veterinarian that performed the observation as a random factor. For FPD, BW was added as a covariate. Data are expressed as mean ± SE. A *P*-value ≤ 0.05 was considered as significant, and *P*-values >0.05 and ≤ 0.10 were considered as a tendency.

## Results

### Mortality

Total mortality PEI (dead + culled) was on average 17.5% (*N* = 56), from which 15.3% occurred within 1 week PEI. The percentage of dead or culled broilers PEI did not show an interaction between EST and feeding strategy (Table 1; *P* ≥ 0.28) nor a main effect of EST (*P* ≥ 0.48) or feeding strategy (*P* ≥ 0.11). Total mortality PEI also did not show an interaction between EST and feeding strategy (*P* = 0.46) nor showed a main effect of EST (*P* = 0.68). However, early feeding tended to result in a lower total mortality PEI compared to delayed feeding (Δ = 6.6%; *P* = 0.06).

**Table 1:** Effect of eggshell temperature (**EST**) during late incubation [≥E17–E19.5; 37.8°C (**control**) or 36.7°C (**lower**)] and/or feeding strategy after hatch [immediate access to feed and water (**early**) or 51–54-h deprivation (**delayed**)] on mortality of broilers during 2 weeks following necrotic enteritis induced at d 21 post hatch (LSmeans ± SEM).

	***N* ^a^**	**Died (%)**	**Culled (%)**	**Total mortality^b^ (%)**
**EST**				
Control	16	9.9	4.9	14.8
Lower	16	15.0	3.1	18.1
SEM		2.75	1.76	3.12
**Feeding strategy**				
Early	16	10.6	2.6	13.1
Delayed	16	14.3	5.4	19.7
SEM		2.75	1.76	3.12
**EST × Feeding strategy**				
Control × Early	8	7.9	2.5	10.4
Control × Delayed	8	11.9	7.3	19.2
Lower × Early	8	13.3	2.6	15.9
Lower × Delayed	8	16.7	3.5	20.2
SEM		3.89	2.49	4.38
***P*** **-values**				
EST		0.56	0.48	0.68
Feeding strategy		0.11	0.39	0.06
EST × Feeding strategy		0.28	0.47	0.46

a *Number of pens*.

b *Total mortality = died + culled*.

### BW Changes

**Figure 1:**
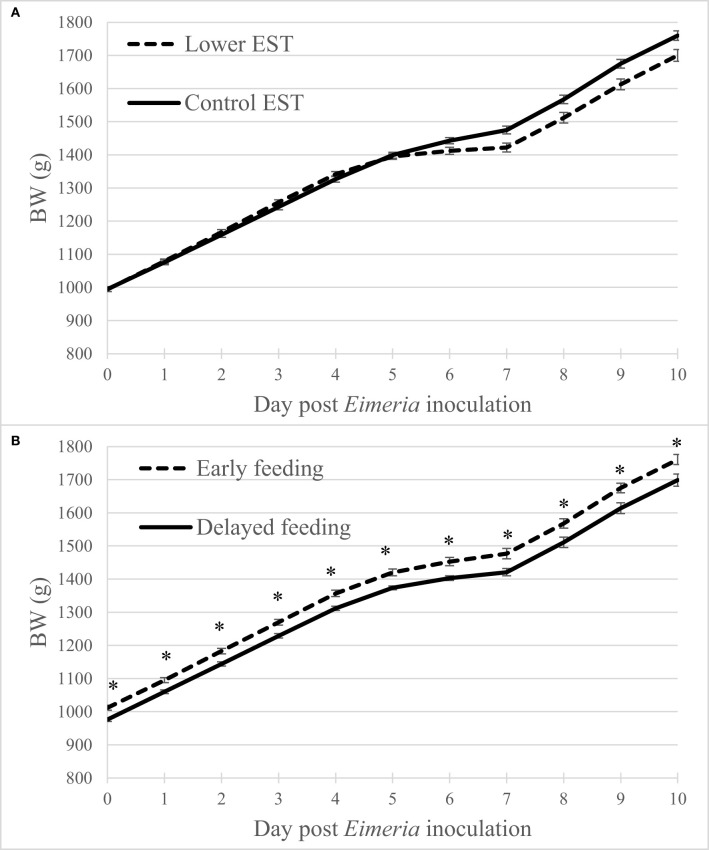
**(A)** Effect of eggshell temperature (**EST**) during late incubation [≥embryonic day 17–19.5; 37.8°C (**control**) or 36.7°C (**lower**)] or **(B)** effect of feeding strategy after hatch [immediate access to feed and water (**early**) or 51–54-h deprivation (**delayed**)] on broiler BW during 10 days post *Eimeria* inoculation (d 21 post hatch) to induce necrotic enteritis (*N* = 16 pens/treatment). An asterisk (*) indicate significant difference (*P* < 0.05) between LSmeans of treatments within day post *Eimeria* inoculation. Error bars indicate standard error.

Regardless of the d PEI, BW did not show an interaction between EST and feeding strategy (*P* = 0.30) nor showed a main effect of EST (Figure 1A; *P* = 0.37). Early feeding resulted in higher BW at all days compared to delayed feeding (Figure 1B; *P* = 0.04). Males had higher BW compared to females at any d PEI (*P* < 0.01; data not shown).

**Figure 2:**
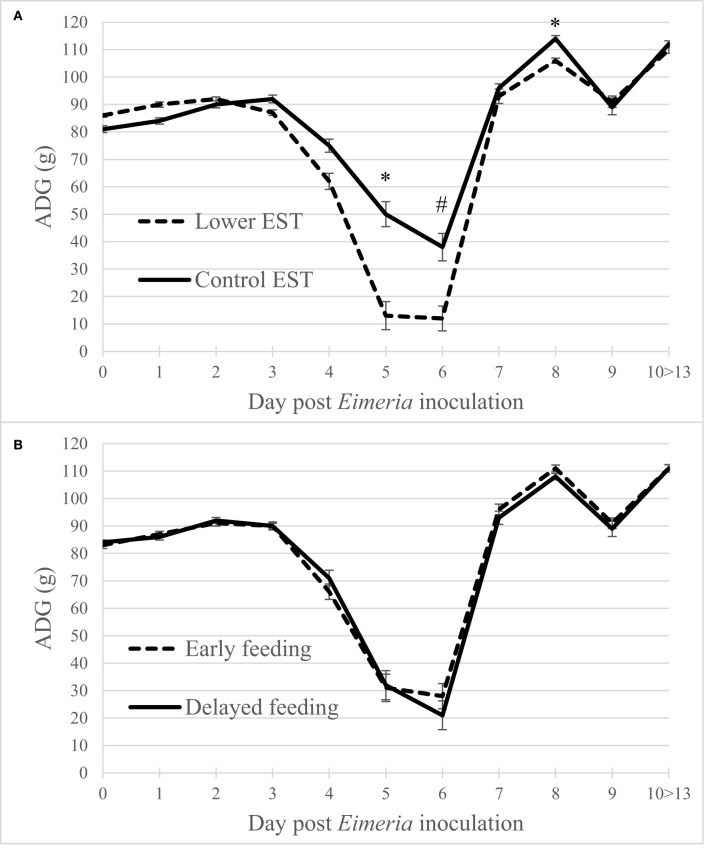
**(A)** Effect of eggshell temperature (**EST**) during late incubation [≥embryonic day 17–19.5; 37.8°C (**control**) or 36.7°C (**lower**)] or **(B)** effect of feeding strategy after hatch [immediate access to feed and water (**early**) or 51–54-h deprivation (**delayed**)] on broiler average daily gain during 13 days post *Eimeria* inoculation (d 21 post hatch) to induce necrotic enteritis (*N* = 16 pens/treatment). An asterisk (*) indicate significant difference (*P* < 0.05) between LSmeans of treatments within day post *Eimeria* inoculation and a hashtag (^#^) indicates s tendency to differ (*P* < 0.10). Error bars indicate standard error.

Regardless of the d PEI, ADG did not show an interaction between EST and feeding strategy (*P* = 0.13) nor a main effect of feeding strategy (Figure 2B; *P* = 0.49). Lower EST resulted in a lower ADG at d 5 and 8 PEI (Figure 2A; *P* = 0.02 both days) and tended to result in a lower ADG at d 6 PEI (*P* = 0.06) compared to control EST. Males had a higher ADG compared to females at any d PEI (*P* < 0.01; data not shown).

### Necrotic Enteritis Lesions

NE lesion severity was worse on d 6 PEI compared to d 7 PEI (Δ = 1.0 score; *P* < 0.01). Neither on d 6 PEI nor on d 7 PEI did severity of NE lesions show an interaction between EST and feeding strategy (Table 2; *P* ≥ 0.31) nor a main effect of EST (*P* ≥ 0.46) or feeding strategy (*P* ≥ 0.43). NE incidence was on average 84.4%, which was similar on d 6 and 7 PEI (*P* = 0.27), and did not show an interaction between EST and feeding strategy (*P* = 0.31) nor show a main effect of EST (*P* = 0.51) or feeding strategy (*P* = 0.14). At d 7 PEI, males tended to have more severe NE lesions compared to females (Δ = 0.4 score; *P* = 0.09), but at d 6 PEI, severity of NE lesions was not different between sexes (*P* = 0.26). NE incidence was not different between sexes (*P* = 0.83).

**Table 2:** Effect of eggshell temperature (**EST**) during late incubation [≥E17–E19.5; 37.8°C (**control**) or 36.7°C (**lower**)] and/or feeding strategy after hatch [immediate access to feed and water (**early**) or 51–54-h deprivation (**delayed**)] on necrotic lesions in the small intestine of broilers at d 6 or 7 post *Eimeria* inoculation (d 21 post hatch) to induce necrotic enteritis (mean ± SE).

	***N* ^a^**	**NE severity (score 0–6)**		**NE incidence^c^ (%)**
		**Day post** ***Eimeria*** **inoculation^b^**		
		**6**	**7**	
**EST**				
Control	16	3.8 ± 0.24	3.0 ± 0.16	82.9 ± 3.05
Lower	16	4.0 ± 0.23	2.8 ± 0.15	85.4 ± 2.87
**Feeding strategy**				
Early	16	3.9 ± 0.22	2.9 ± 0.14	87.7 ± 2.65
Delayed	16	3.9 ± 0.25	2.8 ± 0.17	80.5 ± 3.24
**EST × Feeding strategy**				
Control × Early	8	3.6 ± 0.34	2.9 ± 0.20	84.2 ± 4.18
Control × Delayed	8	4.0 ± 0.33	3.0 ± 0.25	81.6 ± 4.45
Lower × Early	8	4.2 ± 0.28	2.9 ± 0.20	91.0 ± 3.24
Lower × Delayed	8	3.8 ± 0.37	2.6 ± 0.23	79.5 ± 4.73
***P*** **-values**				
EST		0.91	0.46	0.51
Feeding strategy		0.87	0.43	0.14
EST × Feeding strategy		0.50	0.31	0.31
Sex		0.26	0.09	0.83

a *Number of pens*.

b *Five broilers per pen per day*.

c *Broilers with lesion scores of 2 or more were classified as NE positive*.

### Coccidiosis

Neither on the day of *Eimeria* inoculation nor on d 6 PEI or d 7 PEI did severity (TMLS) or incidence (%) of coccidiosis show an interaction between EST and feeding strategy (Table 3; *P* ≥ 0.34) nor a main effect of EST (*P* ≥ 0.23) or feeding strategy (*P* ≥ 0.58). At d 14 PEI, a lower EST resulted in a lower coccidiosis severity (Δ = 0.3 TMLS; *P* = 0.01) and tended to result in a lower coccidiosis incidence (Δ = 15.7%; *P* = 0.09) compared to control EST. Severity and incidence of coccidiosis at d 14 PEI both did not show an interaction between EST and feeding strategy (*P* ≥ 0.30) nor a main effect of feeding strategy (*P* ≥ 0.20). There was no difference in severity or incidence of coccidiosis between sexes (*P* ≥ 0.27).

**Table 3:** Effect of eggshell temperature (**EST**) during late incubation [≥E17–E19.5; 37.8°C (**control**) or 36.7°C (**lower**)] and/or feeding strategy after hatch [immediate access to feed and water (**early**) or 51–54-h deprivation (**delayed**)] on coccidiosis of broilers at d 0, 6, 7, or 14 post *Eimeria* inoculation (d 21 post hatch) to induce necrotic enteritis (mean ± SE).

	***N* ^c^**	**Coccidiosis severity (TMLS**^d^ **0–12)**				**Coccidiosis incidence^e^ (%)**			
		**Day post** ***Eimeria*** **inoculation^f^**				**Day post** ***Eimeria*** **inoculation**^f^			
		**0**	**6**	**7**	**14**	**0**	**6**	**7**	**14**
**EST**									
Control	16	2.2 ± 0.18	3.8 ± 0.24	2.8 ± 0.20	0.9 ± 0.10^a^	85.3 ± 4.17	92.1 ± 3.09	92.1 ± 3.09	70.0 ± 5.1
Lower	16	1.9 ± 0.21	4.1 ± 0.23	3.2 ± 0.21	0.6 ± 0.07^b^	90.6 ± 5.15	96.1 ± 2.21	96.0 ± 2.29	54.3 ± 5.53
**Feeding strategy**									
Early	16	2.2 ± 0.23	3.8 ± 0.22	3.0 ± 0.20	0.8 ± 0.10	86.0 ± 5.28	94.8 ± 2.53	94.8 ± 2.53	55.6 ± 5.52
Delayed	16	2.0 ± 0.17	4.0 ± 0.25	3.0 ± 0.21	0.9 ± 0.08	87.7 ± 4.13	93.4 ± 2.84	93.2 ± 2.96	68.8 ± 5.18
**EST × Feeding strategy**									
Control × Early	8	2.4 ± 0.26	3.7 ± 0.33	2.8 ± 0.30	1.0 ± 0.16	86.1 ± 5.76	94.7 ± 3.62	92.1 ± 4.37	67.5 ± 7.41
Control × Delayed	8	2.1 ± 0.24	3.8 ± 0.35	2.9 ± 0.26	1.0 ± 0.12	84.4 ± 6.02	89.5 ± 4.98	92.1 ± 4.37	72.5 ± 7.06
Lower × Early	8	1.6 ± 0.40	4.0 ± 0.30	3.2 ± 0.26	0.5 ± 0.10	85.7 ± 13.23	94.9 ± 3.53	97.4 ± 2.53	43.9 ± 7.75
Lower × Delayed	8	2.0 ± 0.24	4.1 ± 0.36	3.1 ± 0.32	0.8 ± 0.10	92.0 ± 5.43	97.4 ± 2.60	94.3 ± 3.92	65.0 ± 7.54
***P*** **-values**									
EST		0.23	0.68	0.34	0.01	0.79	0.46	0.35	0.09
Feeding strategy		0.87	0.79	0.99	0.38	0.58	0.92	0.59	0.20
EST × Feeding strategy		0.34	0.85	0.53	0.30	0.80	0.61	0.57	0.38
Sex		0.31	0.57	0.90	0.39	0.77	0.84	0.27	0.54

a,b *Means within a column and factor lacking a common superscript differ (P < 0.05)*.

c *Number of pens*.

d *TMLS = Total mean lesion score. Sum of coccidiosis score for E. acervulina, E. maxima, and E. tenella*.

e *Broilers with a TMLS of 1 or more were classified as coccidiosis positive*.

f *In total, 100, 153, 150, and 161 broilers for d 0, 6, 7, and 14, respectively*.

### Dysbacteriosis

Neither on the day of *Eimeria* inoculation nor on d 14 PEI did severity or incidence (%) of dysbacteriosis show an interaction between EST and feeding strategy (Table 4; *P* ≥ 0.34) nor a main effect of EST (*P* ≥ 0.40) or feeding strategy (*P* ≥ 0.25). There was no difference in severity or incidence of dysbacteriosis between sexes on either dissection day (*P* ≥ 0.63).

**Table 4:** Effect of eggshell temperature (**EST**) during late incubation [≥embryonic days 17–19.5; 37.8°C (**control**) or 36.7°C (**lower**)] and/or feeding strategy after hatch [immediate access to feed and water (**early**) or 51–54-h deprivation (**delayed**)] on dysbacteriosis of broilers at d 0 or 14 post *Eimeria* inoculation (d 21 post hatch) to induce necrotic enteritis (mean ± SE).

	***N* ^a^**	**Dysbacteriosis severity (score 0–10)**		**Dysbacteriosis incidence^b^ (%)**	
		**Day post** ***Eimeria*** **inoculation**^c^		**Day post** ***Eimeria*** **inoculation**^c^	
		**0**	**14**	**0**	**14**
**EST**					
Control	16	2.9 ± 0.15	2.8 ± 0.15	33.8 ± 5.80	33.8 ± 5.29
Lower	16	2.8 ± 0.19	2.9 ± 0.14	21.9 ± 7.31	35.8 ± 5.33
**Feeding strategy**					
Early	16	2.7 ± 0.19	2.9 ± 0.15	25.6 ± 6.65	35.8 ± 5.33
Delayed	16	3.0 ± 0.14	2.9 ± 0.14	33.3 ± 6.33	33.8 ± 5.29
**EST × Feeding strategy**					
Control × Early	8	2.7 ± 0.21	2.9 ± 0.22	27.8 ± 7.47	37.5 ± 7.65
Control × Delayed	8	3.2 ± 0.19	2.8 ± 0.21	40.6 ± 8.86	30.0 ± 7.25
Lower × Early	8	2.7 ± 0.39	2.8 ± 0.21	14.3 ± 13.23	34.1 ± 7.41
Lower × Delayed	8	2.8 ± 0.21	3.0 ± 0.19	24.0 ± 8.54	37.5 ± 7.65
***P*** **-values**					
EST		0.44	0.40	0.88	0.98
Feeding strategy		0.25	0.30	0.94	0.66
EST × Feeding strategy		0.91	0.34	0.44	0.53
Sex		0.95	0.95	0.63	0.89

a *Number of pens*.

b *Broilers with a score of 3 or more were classified as dysbacteriosis positive*.

c *In total, 100 and 161 broilers for d 0 and 14, respectively*.

### Oocyst Shedding

**Figure 3:**
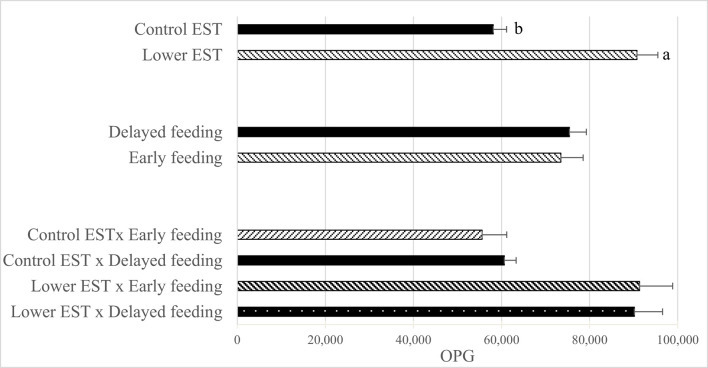
Effect of eggshell temperature (**EST**) during late incubation [≥embryonic day 17–19.5; 37.8°C (**control**) or 36.7°C (**lower**)] and/or feeding strategy after hatch [immediate access to feed and water (**early**) or 51–54-h deprivation (**delayed**)] on *E. Maxima* oocytes per gram feces (**OPG**) at d 8 post *Eimeria* inoculation (d 21 post hatch) to induce necrotic enteritis (*N* = 16 pens/treatment). (a,b) Least squares means within a factor lacking a common superscript differ (*P* < 0.05). Error bars indicate standard error.

Neither 1 day before *Eimeria* inoculation nor at d 5 PEI was *E. maxima* OPG found in any of the pens. *E. maxima* OPG at d 8 PEI did not show an interaction between EST and feeding strategy (Figure 3; *P* = 0.79) nor a main effect of feeding strategy (*P* = 0.87), but a lower EST resulted in a higher *E. maxima* OPG at d 8 PEI compared to control EST (Δ = 32,638 ± 4,117 OPG; *P* < 0.01).

### Natural Antibodies

IgM NAb titer on the day of inoculation, d 6 PEI, and d 14 PEI showed neither an interaction between EST and feeding strategy (Table 5; *P* ≥ 0.14) nor a main effect of EST (*P* ≥ 0.75) or feeding strategy (*P* ≥ 0.74). At d 6 PEI, males tended to show higher IgM NAb titer compared to females (Δ = 0.5 titer; *P* = 0.06), and at d 14 PEI, IgM NAb titer was higher in males compared to females (Δ = 0.8 titer; *P* < 0.01).

**Table 5:** Effect of eggshell temperature (**EST**) during late incubation [≥embryonic day 17–19.5; 37.8°C (**control**) or 36.7°C (**lower**)] and/or feeding strategy after hatch [immediate access to feed and water (**early**) or 51–54-h deprivation (**delayed**)] on IgM and IgY natural antibody (NAb) titer against keyhole limpet hemocyanin of broilers at d 0, 6, or 14 post *Eimeria* inoculation (d 21 post hatch) to induce necrotic enteritis (LSmeans ± SEM).

	***N* ^c^**	**IgM**			**IgY**		
		**Day post** ***Eimeria*** **inoculation^d^**			**Day post** ***Eimeria*** **inoculation**^d^		
		**0**	**6**	**14**	**0**	**6**	**14**
**EST**							
Control	16	1.6	2.6	4.1	0.9	1.1	3.0
Lower	16	1.6	2.5	4.1	0.7	1.1	3.1
SEM	0.2	0.2	0.2	0.2	0.1	0.3	
**Feeding strategy**							
Early	16	1.5	2.6	4.2	0.8	1.2	3.1
Delayed	16	1.7	2.6	4.1	0.8	1.1	3.1
SEM	0.3	0.2	0.2	0.2	0.1	0.3	
**EST × Feeding strategy**							
Control × Early	8	1.6	2.3	4.4	1.0	1.0^a,b^	3.0
Control × Delayed	8	1.5	3.0	3.9	0.8	1.3^a,b^	3.1
Lower × Early	8	1.4	2.9	4.0	0.5	1.4^a,b^	3.1
Lower × Delayed	8	1.9	2.2	4.3	0.9	0.8^a,b^	3.0
SEM	0.4	0.3	0.3	0.2	0.2	0.4	
***P*** **-values**							
EST		0.99	0.75	0.93	0.41	0.71	0.90
Feeding strategy		0.91	0.99	0.74	0.60	0.42	0.98
EST × Feeding strategy		0.39	0.17	0.14	0.28	0.04	0.70
Sex		0.69	0.06	< 0.01^e^	0.78	0.87	0.71

a,b *Least squares means within a column and factor lacking a common superscript differ (P < 0.05)*.

c *Number of pens*.

d *In total 48, 51, and 57 broilers for d 0, 6, and 14, respectively*.

e *Titers 4.5 and 3.7 (±0.20) for males and females, respectively*.

IgY NAb titer at d 6 PEI showed an interaction between EST and feeding strategy (Table 5; *P* = 0.04). Early feeding resulted in a higher IgY NAb titer compared to delayed feeding if incubated at a lower EST (Δ = 0.6 titer), but not if incubated at the control EST. IgY NAb titer on the day of *Eimeria* inoculation and at d 14 PEI did not show an interaction between EST and feeding strategy (*P* ≥ 0.28) nor a main effect of EST (*P* ≥ 0.41) or feeding strategy (*P* ≥ 0.60). There was no difference in IgY NAb titer between sexes at any dissection day (*P* ≥ 0.71).

### Footpad Dermatitis

FPD incidence at d 6 PEI showed an interaction between sex and feeding strategy (Table 6; *P* = 0.04). Early-fed females had lower FPD incidence compared to delayed-fed females (Δ = 13.0%; data not shown), while early- and delayed-fed males did not differ in FPD incidence at this day. FPD incidence at d 6 PEI did not show an interaction between EST and feeding strategy (*P* = 0.68) nor a main effect of EST (*P* = 0.31). FPD incidence at d 7 PEI did not show an interaction between EST and feeding strategy (*P* = 0.73) nor a main effect of feeding strategy (*P* = 0.14). FPD incidence at d 7 PEI tended to be higher in control EST compared to lower EST (Δ = 15.5%; *P* = 0.09). On the day of *Eimeria* inoculation and at d 14 PEI, FPD incidence did not show an interaction between EST and feeding strategy (*P* ≥ 0.56) nor a main effect of EST (*P* ≥ 0.13) or feeding strategy (*P* ≥ 0.17). There was no difference in FPD incidence between sexes at the day of *Eimeria* inoculation and at d 7 PEI or d 14 PEI (*P* ≥ 0.20).

**Table 6:** Effect of eggshell temperature (**EST**) during late incubation [≥embryonic days 17–19.5; 37.8°C (**control**) or 36.7°C (**lower**)] and/or feeding strategy after hatch [immediate access to feed and water (**early**) or 51–54 h deprivation (**delayed**)] on average footpad dermatitis (FPD) incidence of broilers at d 0, 6, 7, or 14 post *Eimeria* inoculation (d 21 post hatch) to induce necrotic enteritis (mean ± SE).

	***N* ^a^**	**FPD (%)** ^b^			
		**Day post** ***Eimeria*** **inoculation**^c^			
		**0**	**6**	**7**	**14**
**EST**					
Control	16	14.9 ± 4.35	19.7 ± 4.57	26.3 ± 5.05	36.3 ± 5.33
Lower	16	0.0 ± 0.00	10.4 ± 3.48	10.8 ± 3.61	21.0 ± 4.52
**Feeding strategy**					
Early	16	2.3 ± 2.30	13.0 ± 3.83	11.7 ± 3.66	21.0 ± 4.52
Delayed	16	16.1 ± 4.91	17.1 ± 4.32	26.0 ± 5.14	36.3 ± 5.33
**EST** **×** **Feeding strategy**					
Control × Early	8	2.8 ± 2.74	18.4 ± 6.29	18.4 ± 6.29	30.0 ± 7.25
Control × Delayed	8	29.0 ± 8.15	21.1 ± 6.61	34.2 ± 7.70	42.5 ± 7.75
Lower × Early	8	0.0 ± 0.00	7.7 ± 4.27	5.1 ± 3.53	12.2 ± 5.11
Lower × Delayed	8	0.0 ± 0.00	13.2 ± 5.48	17.1 ± 6.37	30.0 ± 7.25
***P*** **-values**					
EST		0.98	0.31	0.09	0.13
Feeding strategy		0.99	0.65	0.14	0.17
EST × Feeding strategy		0.99	0.68	0.73	0.56
Sex		0.20	0.42	0.68	0.50
Body weight		0.48	0.82	0.32	0.11
Sex × Feeding strategy		n.a.	0.04	n.a.	n.a.

a *Number of pens*.

b *Broilers with a score of 1 or 2 were classified as FPD positive*.

c *In total 99, 153, 150, and 161 broilers for d 0, 6, 7, and 14, respectively*.

## Discussion

The aim of this study was to investigate whether or not broiler resilience could be affected by EST during late incubation and/or by feeding strategy immediately post hatch. Resilience was defined in this study as the capability to absorb environmental disturbances and reorganize with minimal loss of function, according to (2). Therefore, NE was induced at d 21 post hatch to model an environmental disturbance, and loss of function was determined by measuring mortality and changes in BW. Disease morbidity measures were also determined, but as discussed in the following paragraphs, it is rather difficult to draw conclusions about resilience based on these morbidity measures.

### Necrotic Enteritis Model

Regardless of treatment, the disturbance model successfully induced NE. After *Eimeria* inoculation, clinical symptoms of the disease were observed and mortality and a severe reduction in ADG occurred. An overall NE incidence of 84.1% was found with NE lesion scores of 3.9 and 2.8 out of 6 at d 6 and 7 PEI, respectively. Additionally, an increase of *E. maxima* oocysts shed in the feces at d 8 PEI was seen. Remarkably, no increase in feces oocysts of *E. acervulina* and *E. mitis* was found, while both species were also inoculated. Likely, the predicted increase in oocyst shedding of these two species remained absent, because oocysts of both species were already found in feces prior to *Eimeria* inoculation. It was shown before that chickens can develop immunity to *Eimeria* (50). However, although the presence of *E. acervulina* and *E. mitis* species prior to *Eimeria* inoculation may have affected the NE disease response, NE was still clearly induced successfully after inoculation of *Eimeria* at d 21.

The NE disease model induced mortality (17.5%) and a clear disturbance pattern in growth performance with a gradual drop in ADG up to 80 g at d 3 to 4 PEI. This makes the current NE disease model suitable to study broiler resilience, because loss of function can be determined through mortality rates and the speed and extension at which broilers dropped their BW. Remarkably, ADG sharply increased thereafter within 1 day back to pre-inoculation levels. Because of this rapid recovery, it is hard to draw conclusions on possible differences in recovery speed. Recovery speed is also regarded as part of resilience, as “reorganization” is part of the resilience definition (2). A different disease model that induces a disease with a slower recovery speed may give better insights in kinetics of reorganization after a disturbance. However, the rapid recovery in the current study may also indicate that broilers at this age are relatively resilient animals in terms of recovery speed. Results from the current study are representative for 4-week-old broilers of an old breeder flock and may help to manage NE, the most common disease in the poultry industry, which occurs mainly around that age (51, 52).

### Interaction EST × Feeding Strategy

This study was the first study to investigate a possible interaction between prenatal thermal conditions and early feeding. It was hypothesized that EST and feeding strategy may interact with each other as EST may for instance affect embryo development in such a way that a newborn chick is better prepared for exogenous feed intake at hatch moment, and consequently, the optimal combination of factors during neonatal life may affect broiler resilience in later life. The hypothesis was not confirmed as no interaction was found in the majority of the parameters. No difference in mortality, BW changes, or disease morbidity parameters were found with the exception of natural antibodies binding to KLH. At d 6 PEI, early-fed broilers had a higher average NAb IgY titer compared to delayed-fed broilers if these broilers were incubated at a lower EST but not if they were incubated at the control EST. At first instance, the finding of only one significant interaction between EST and feeding strategy might not seem a very strong result, because the interaction was not consistently shown at other ages and occurred only in IgY but not in IgM isotype. However, the interaction was found at d 6 PEI, at which the worst diseased state seemed to occur and may therefore only be manifested in a severely diseased state. The fact that differences were only found in the IgY isotype and not IgM may be explained by the inflammatory immune responses that occur during NE, whereas isotype IgY antibodies are strongly involved in the release of inflammatory mediators. So, the current finding of an interaction between EST and feeding strategy on the NAb IgY titer at d 6 PEI is a minor indication that these two factors may interfere on a broiler's immune system, and as of yet, it is unknown whether or not EST during late incubation interacts with early moment of feeding post hatch. For future studies, it might be interesting to investigate other EST patterns rather than the lower EST during late incubation from the current study. Prior to the current study, we hypothesized that EST may perhaps affect embryo development in such a way that the neonatal chick could benefit optimally from immediate access to feed and water after hatch, for instance, *via* altering intestinal morphology or digestive enzyme activities. However, the lower EST during late incubation in the current study may not have stimulated these parts of embryogenesis such that the hypothesized mechanism of interaction did not occur. This is supported by findings that a lower EST during late incubation impairs embryo development (13, 16, 35) instead of improving it (15, 53), or perhaps, the only effect was a slower development of the embryo (54).

### EST

The current study showed that broiler resilience during later life appears to be modulated by EST during late incubation. Broilers incubated at a lower EST of 36.7°C during late incubation seemed to have impaired resilience, as they showed more difficulties coping with NE compared to broilers incubated at a control EST of 37.8°C. This impairment in resilience was especially shown by the ADG pattern. In the first 4 days PEI, ADG was similar between both EST groups and was not yet affected to a large extent by *Eimeria* inoculation. During the following 3 days thereafter, ADG was declining in both EST treatments, but the decline was larger in the lower-EST group (from ~90 to 10 g/day) compared to the control EST group (~90–40 g/day), with a significant difference at d 5 and 8 and a tendency at d 6 PEI (*P* = 0.06). Besides, the lower-EST group reached their lowest ADG after *Eimeria* inoculation 1 day earlier and maintained at this low level 1 day longer compared to the control EST group. Lower-EST incubated broilers also seemed to shed more oocysts in their feces, indicated by a higher *E. maxima* OPG at d 8 PEI. To the author's knowledge, no other studies were published yet that investigated the effect of a lower incubation temperature during late incubation on broiler resilience. Nevertheless, the current finding that EST might affect broiler resilience in later life is somewhat supported by two other studies, which found a higher mortality rate during the growing phase in broilers incubated at a lower late-incubation temperature (13, 55).

Meanwhile, lower-EST incubated broilers did not show a lower incidence or severity of morbidity parameters compared to control EST broilers. In fact, lower-EST broilers had less severe clinical signs of coccidiosis at 2 weeks PEI. This could be interpreted as a faster recovery of lower-EST broilers, indicating improved resilience, and this may appear contradictory to conclusions based on ADG. However, incidence and severity of morbidity can change rapidly over time and can vary even between consecutive days (56, 57). For instance, in the current study, the NE lesion score was significantly higher on d 6 PEI compared to d 7 PEI. It is possible that the development of morbidity over time varied between treatment groups. In such cases, conclusions about broiler resilience by looking at morbidity parameters at separate time points should be drawn with care, because it remains unclear what phase of functional loss was studied.

### Feeding Strategy

Early feeding tended (*P* = 0.06) to lower total mortality after NE induction compared to delayed feeding (Δ = 6.6%). Death can be regarded as the ultimate “loss of function” as these broilers were unable to “reorganize” after the disturbance. Therefore, this tendency is an indication that resilience in later life might be improved by early feeding.

Meanwhile, no difference was found between early- and delayed-fed broilers in the number of shed *Eimeria* oocysts and severity or incidence of NE lesions, coccidiosis, and dysbacteriosis. As discussed before, morbidity measures can provide useful information to study disease severity and incidence at specific time points. These morbidity measures may, however, be somewhat misleading when studying resilience, because pathogenesis can rapidly change over time and can vary significantly between consecutive days, meaning that essential information about loss of function can be missed if measurements are performed only at specific time points. A more convincing support of possible effects of feeding strategy on resilience was expected to be found in changes in BW after NE. Despite consistently higher BWs of early-fed broilers after onset of NE, the difference in BW between early- and delayed-fed broilers remained the same over time. Additionally, loss of ADG after onset of NE was similar between both feeding groups. This indicates that feeding strategy had no effect on the loss of function, and therefore, broiler resilience to NE in the 4th week of life seems unaffected. Perhaps no differences in changes in BW were found because only surviving broilers were included in these analyses. The delayed-feeding treatment tended to have higher mortality, and likely, dying broilers had the largest losses in BW. However, additional analysis on BW change, including the broilers that died, showed comparable results to those currently presented.

Feeding strategy might have an effect on broiler resilience that is more clearly pronounced during the first 2–3 weeks post hatch. For instance, authors in (30) also induced NE, but instead they inoculated *Eimeria* at d 9 post hatch, and they showed that early-fed broilers had increased resilience to NE in terms of growth performance. In the current study, NE was induced at d 21 post hatch, and resilience was subsequently determined during the 4th week of life. It could be that delayed-fed broilers showed compensatory development, meaning that potential differences between early- and delayed-feeding strategies were not clearly pronounced in changes in BW. Studies showed that although full compensation was not observed, delayed-fed broilers do indeed show compensatory BW gain to some extent (21, 27, 28, 30, 58). Moreover, the majority of differences in immune response that were found between early- and delayed-fed broilers seems to diminish after 3 weeks post hatch (24, 25, 59). Hollemans et al. (59) also found no indication of different later-life resilience between early- and delayed-fed broilers. In the latter study, early and 72-h-delayed-fed broilers of a prima breeder flock were challenged by housing them in poor sanitary conditions.

Early-fed females had a lower incidence of FPD at d 6 PEI compared to delayed-fed females (Δ = 13.0%; *P* = 0.04), whereas this difference was not found in males. Prevalence of FPD is predisposed by wet litter (60, 61), and wet litter can be a result of abnormal fecal droppings caused by intestinal problems. In such a case, a lower incidence of FPD in early-fed females could be an indirect indicator of improved resilience. However, in the current study, no indications of a difference in gut health between early- and delayed-fed broilers were found as coccidiosis and dysbacteriosis scores were similar between both treatments. Moreover, the difference in FPD was not very consistent as it was observed only on a single collection moment and only in females. Nevertheless, several other studies also found similar indications that early feeding, as a factor of on-farm hatching, may reduce FPD as well as mortality rate during grow-out (62–65). It should be noted though that effects of on-farm hatching cannot be attributed only to early feeding but may be confounded with other factors such as transportation of eggs instead of day-old chicks or EST during the last days of incubation on the farm,. Perhaps early feeding acts on FPD and mortality *via* a mechanism that is not expressed in BW changes. For instance, early feeding may alter broiler activity levels, drinking behavior, or nutritional needs, which all are factors that can also affect litter moisture content (66). Future studies could perhaps investigate which predisposing factor altered FPD incidence between early- and delayed-fed broilers and thereby gather new insights on possible later-life effects of early feeding.

## Conclusion

It can be concluded that EST during late incubation did not interact with feeding strategy on broiler resilience to NE at 4 weeks of age in broilers of an old breeder flock. We found indications that both a lower EST of 36.7°C during late incubation and 51–54-h delayed feeding each may impair later-life resilience compared to a control EST of 37.8°C and immediate access to feed and water after hatch. However, differences between treatments were not manifested consistently in the multiple parameters that were measured, and therefore, these conclusions are drawn with some restraint.

## Data Availability Statement

The raw data supporting the conclusions of this article will be made available by the authors, without undue reservation.

## Ethics Statement

The animal study was reviewed and approved by Governmental Commission on Animal Experiment, The Hague, The Netherlands, approval number: 2018.W-0020.001 and Ethical Committee of Poulpharm, Belgium, approval number P19034-FP.

## Author Contributions

HW conducted the study, analyzed the data, and wrote the main manuscript. All authors conceived and designed the study, provided substantive input, and contributions to manuscript revision.

## Funding

This study was part of a Ph.D. project, at Wageningen University & Research, on improving broiler resilience by adjusting neonatal conditions which was funded by HatchTech B.V. The funder had no further role in the design of the study and collection, analysis, and interpretation of data and in writing the manuscript.

## Conflict of Interest

HW, CP, and IR-R were employees of HatchTech at the time of the study. JS was employed by company Poulpharm BVBA. The remaining authors declare that the research was conducted in the absence of any commercial or financial relationships that could be construed as a potential conflict of interest.

## Publisher's Note

All claims expressed in this article are solely those of the authors and do not necessarily represent those of their affiliated organizations, or those of the publisher, the editors and the reviewers. Any product that may be evaluated in this article, or claim that may be made by its manufacturer, is not guaranteed or endorsed by the publisher.

## Abbreviations

ADG: average daily gain
E: embryonic day
EST: eggshell temperature
FPD: footpad dermatitis
KLH: keyhole limpet hemocyanin
Nab: natural antibodies
NE: necrotic enteritis
OPG: oocysts per gram of feces
PEI: post *Eimeria* inoculation
TMLS: total mean lesion score.
